# Cannabis and tobacco use prior to pregnancy and subsequent offspring birth outcomes: a 20-year intergenerational prospective cohort study

**DOI:** 10.1038/s41598-021-95460-2

**Published:** 2021-08-19

**Authors:** Lindsey A. Hines, Elizabeth A. Spry, Margarita Moreno-Betancur, Hanafi Mohamad Husin, Denise Becker, Melissa Middleton, Jeffrey M. Craig, Lex W. Doyle, Craig A. Olsson, George Patton

**Affiliations:** 1 Centre for Academic Mental Health, Population Health Sciences Institute, University of Bristol, Bristol, UK; 2 MRC Integrative Epidemiology Unit, Population Health Sciences Institute, University of Bristol, Bristol, UK; 3 Centre for Social and Early Emotional Development, Faculty of Health, Deakin University, Melbourne, Australia; 4 Centre for Adolescent Health, Murdoch Children’s Research Institute, Melbourne, Australia; 5 University of Melbourne, Melbourne, Australia; 6 Biostatistics Unit, Faculty of Health, Deakin University, Melbourne, Australia; 7 Clinical Epidemiology and Biostatistics Unit, Murdoch Children’s Research Institute, Melbourne, Australia; 8 Centre for Molecular and Medical Research, Deakin University School of Medicine, Geelong, Australia; 9 Department of Obstetrics and Gynaecology, The Royal Women’s Hospital, University of Melbourne, Melbourne, Australia; 10 Clinical Sciences, Murdoch Children’s Research Institute, Melbourne, Australia; 11 Department of Paedatrics, University of Melbourne, Melbourne, Australia

**Keywords:** Psychology, Risk factors

## Abstract

There is increasing evidence that the life-course origins of health and development begin before conception. We examined associations between timing and frequency of preconception cannabis and tobacco use and next generation preterm birth (PTB), low birth weight (LBW) and small for gestational age. 665 participants in a general population cohort were repeatedly assessed on tobacco and cannabis use between ages 14–29 years, before pregnancy. Associations were estimated using logistic regression. Preconception parent (either maternal or paternal) daily cannabis use age 15–17 was associated with sixfold increases in the odds of offspring PTB (aOR 6.65, 95% CI 1.92, 23.09), and offspring LBW (aOR 5.84, 95% CI 1.70–20.08), after adjusting for baseline sociodemographic factors, parent sex, offspring sex, family socioeconomic status, parent mental health at baseline, and concurrent tobacco use. There was little evidence of associations with preconception parental cannabis use at other ages or preconception parental tobacco use. Findings support the hypothesis that the early life origins of growth begin before conception and provide a compelling rationale for prevention of frequent use during adolescence. This is pertinent given liberalisation of cannabis policy.

## Introduction

Birth status profoundly affects health across the life-course^1^. Prematurity is not only a leading cause of neonatal death but those who survive have greater risks for neurodevelopmental disabilities in later childhood, cardiovascular and metabolic diseases in later life, and face greater socio-economic disadvantage^2–5^. Low birth weight is commonly associated with prematurity and similarly predicts later life cardiometabolic risks^6,7^ and neurodevelopmental disorders^8^. Despite improvements in antenatal care, rates of low birthweight and preterm birth remain high in all countries, affecting over one-in-ten births across the globe^9,10^.

The causes of poor birth outcomes remain unknown in the great majority of cases, though a number of socio-demographic, behavioural and medical risk factors have been identified^11^. Some of the most clearly documented risks are around maternal substance use.

Maternal antenatal tobacco use predicts both prematurity and low birthweight, and for that reason has become a prevention target in pregnancy^12,13^. Frequency of substance use is increasingly recognised as an important factor in determining harms^14,15^, and small-sample studies of maternal tobacco use have indicated a dose–response relationship between frequency or amount of tobacco used during pregnancy, and reduced birth weight^16,17^. Antenatal cannabis use has been less studied but is linked to poor birth outcomes^18–20^, although recent research on cannabis use during pregnancy has been critiqued for failing to consider frequency or timing of use^21^. Given that use in pregnancy is increasingly common^22–24^, with shifts in legalisation potentially increasing its availability, maternal cannabis use is also now attracting policy attention^25–28^.

Few studies of antenatal substance use have considered use prior to pregnancy, despite a growing number of reasons to do so. Both tobacco and cannabis use most commonly begin in adolescence, with rates seen to peak in young adulthood prior to median ages of first parenthood^29,30^. In almost all instances, antenatal tobacco and cannabis use are thus a continuation of use from before pregnancy. Women commonly reduce use of substances on recognition of the pregnancy; an event that typically occurs at 6–8 weeks of gestation, well after the major programming events in early pregnancy. Thus, preconception use may affect birth outcomes even when maternal use ceases with recognition of pregnancy. Equally significant is research outlining mechanisms of intergenerational inheritance through the effects of substance use on parental gametes prior to pregnancy^5^, which is not limited to effects from maternal use. Findings from animal studies suggest that tobacco and cannabis have the potential to alter patterns of methylation in paternal gametes conferring risks for offspring health and development^31–35^. Such findings raise a further possibility that exposure of parental gametes to substances such as tobacco and cannabis at times of sensitivity might affect epigenetic marks and subsequent offspring development, even if maternal use ceases before pregnancy^36,37^.

The present analysis uses data from the 2000 Stories Victorian Adolescent Health Cohort Study (VAHCS) and Victorian Intergenerational Health Cohort Study (VIHCS) to prospectively examine associations between preconception parent (mothers and fathers) tobacco and cannabis use, and offspring birth outcomes of gestational age, birth weight, and being born small for gestational age.

### Aims

1. Examine the extent to which preconception smoking and cannabis exposure might predict offspring birth outcomes.
2. Explore whether effects differ by frequency of use or timing.
3. Explore whether associations observed are robust to adjustment for other adolescent risk factors.

## Methods

### Design and participants

Pre-pregnancy exposure data derive from the VAHCS, a ten-wave cohort study of health in young people living in the state of Victoria, Australia, that commenced in August 1992 (methods outlined in detail in previous publications^38^). Participants’ parents or guardians provided informed written consent at VAHCS recruitment. At baseline, a representative sample of the Victorian population of adolescents in year 9 at school was recruited and subsequently reviewed at a further five 6-month intervals during adolescence (waves 2–6, mean ages 15.4–17.4 years). From a total intended sample of 2032 students, 1943 (95.6%) participated at least once during the first six (adolescent) waves. Participants were then assessed three times in early adulthood at waves 7, 8, and 9 (respective mean ages 20.7 years, 24.1 years, and 29.1 years).

**Figure 1:**
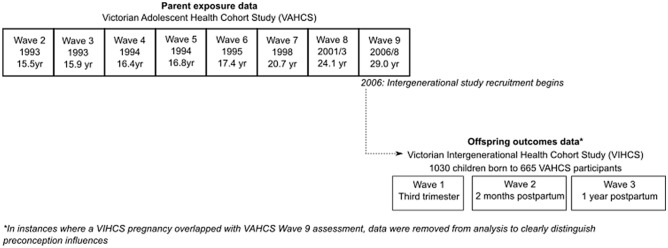
Study design for parent exposure and offspring outcomes.

VIHCS is a longitudinal, prospective cohort study of preconception predictors of early child health and development (methodology and sample characteristics of parents reported previously^39^). It is the intergenerational arm of the established VAHCS cohort. Study members still active in the VAHCS between 2006 (wave 9) (N = 1671) were screened at six-monthly intervals for pregnancies via SMS, email, and phone calls. Participants reporting a pregnancy or recently born infant were invited to complete telephone interviews in trimester three (VIHCS wave 1), 2 months’ postpartum (VIHCS wave 2) and 1 year postpartum (VIHCS wave 3) for every child born during screening. Participants reporting more than one child born during screening were invited to participate with all eligible children. A total of 665 male and female VAHCS study members participated in VIHCS with 1030 offspring; male VAHCS study members reported on the pregnancy of their partners. See Fig. 1, and Supplementary Figure 1 for sample flow chart.

### Ethics and role of funders

Data collection protocols for both studies were approved by the Human Research Ethics Committee at the Royal Children’s Hospital, Melbourne, Australia (approval number 26032). The study was performed in accordance with relevant guidelines and regulations. None of the funders had any role in the study design, collection, management or interpretation of data.

### Measures

#### Frequency of parent tobacco use

Use of tobacco at waves 2–9 of the VAHCS was measured by participant self-report of being a non-smoker/ex-smoker, an occasional smoker (not in past week), a light smoker (< 6 days a week), a medium smoker (6/7 days a week, but ≤ 10 cigarettes a day) or a heavy smoker (6/7 days a week, but > 10 cigarettes a day). Exposures were re-categorised to no use/ex-smoker (reference category), occasional/light tobacco use, and daily tobacco use (combining medium and heavy daily use). Separate summary measures of tobacco use frequency were derived for ages 15–17 years (waves 2–6), 20–24 years (waves 7–8), and age 29 years (wave 9; closest to conception), based on the highest reported level of tobacco use during the relevant time period.

#### Frequency of parent cannabis use

Use of cannabis at VAHCS waves 2–9 was measured through participant self-report of never using cannabis, no use within the previous 6 months, use a few times a year, monthly use, weekly use, or daily use, which was then collapsed to no use within previous 6 months (including never using cannabis), occasional/weekly use (encompassing a few times, monthly and weekly use), and daily use. It should also be noted that after age 17, the period of time covered by this variable was extended to 1 year. Separate summary variables for cannabis use frequency were derived for ages 15–17 years (waves 2–6), 20–24 years (waves 7–8), and 29 years (wave 9; closest to conception), based on the highest reported level of cannabis use during the relevant time period.

#### Outcome measures

Offspring gestational age at birth (completed weeks) and birth weight (kg) were self-reported by parent participants. To derive binary outcomes, offspring were classified as preterm births (PTB) if they were born at less than 37 completed weeks of gestation, and were classified as low birth weight (LBW) if weighing < 2500 g when born. Birthweight Z-scores were calculated relative to the British Growth Reference^40^; offspring were categorised as small for gestational age (SGA) if birthweight was less than the 10th percentile for gestational age (Z-score < − 1.28 SD).

#### Covariates

Offspring sex was determined through parental self report after birth. For all other covariates, parents of the offspring reported these data during their participation in VAHCS. Parent sex was determined through self-report. Family socio-economic status (SES) was calculated by home postcode at study entry using the Index of Relative Socio-economic Disadvantage (IRSD) from the Australian Bureau of Statistics Socio-Economic Index for Areas (SEIFA), then dichotomized so that scores up to the 20th percentile indicated that participants were relatively disadvantaged. Between ages 15–24, VAHCS participants were asked to report on their parents (offspring grandparents) education level, with response options categorised as high school not completed/high school completed/university degree, and their parents (offspring grandparents) smoking status, with response options dichotomised to never or occasionally/most days or every day. For both variables the highest reported level for either parent at any time point was used to determine the status of these summary variables.

Parent mental health at baseline was assessed during VAHCS participation using the revised Clinical Interview Schedule (CIS-R)^41^ at age 15–17. The CIS-R is a branched psychiatric interview designed to assess symptoms of depression and anxiety in non-clinical populations. The total scores on the CIS-R were dichotomized so that scores ≥ 12 indicated a mixed depression-anxiety state at a lower threshold than syndromes of major depression and anxiety disorder, but where clinical intervention would be appropriate^41^.

### Analysis

Data were analysed in Stata 15. We report the crude prevalence of each birth outcome (PTB, LBW and SGA) by frequency of preconception substance use and antenatal tobacco/cannabis exposure (confidence intervals provided as data are imputed). Logistic regression analyses were conducted for each birth outcome measure, to examine the relationship with each exposure (parental cannabis or tobacco use) at 15–17 years, 20–24 years, and 29 years. Robust (Huber–White) standard errors were used in all analyses to account for clustering by family as more than one offspring within the sample could be born from the same parent.

Multivariable models are adjusted for offspring sex, parent sex, family SES, grandparent education level, grandparent smoking status, parent mental health at age 15–17, frequency of parent use of the substance at previous ages (where appropriate), and concurrent use of cannabis for models where tobacco is the exposure and vice versa. In supplementary sensitivity analyses, we repeated all regression analyses (a) with continuous outcome measures using linear regression and (b) further adjusted for periconceptional/antenatal cannabis/tobacco use.

#### Multiple imputation

Most offspring had available data on at least one wave of parent preconception substance use. Missing data in all analysis variables (exposures measured at each of waves 2–9, outcomes, covariates) were addressed through multiple imputation using fully conditional specification—moving time window (FCS-MTW) method^42^: a series of univariate regression models which impute each incomplete variable sequentially given all other variables. Each conditional model included all other outcomes and covariates, as well as exposures measured at the same and adjacent waves as predictors. Maternal weight before pregnancy, maternal high blood pressure/pre-eclampsia, gestational diabetes, primiparae and multiple births (both associated with incomplete offspring birth outcome variables), and highest level of parent education (associated with being missing data on the exposure and outcome variables) were included as auxiliary variables. Summary variables for parent frequency of tobacco and cannabis use were derived following multiple imputation. Estimates were obtained by pooling results across 65 imputed datasets using Rubin’s rules.

#### Population attributable fraction (PAF)

See Appendix 1 for methodology for calculation of the population attributable fraction.

## Results

### Sample description

Table 1 summarises the estimated frequency of offspring outcomes, preconception exposures, antenatal tobacco/cannabis use and potential confounders in the imputed data. See Supplementary Table 1 for proportion of missing data of study variables in the observed data. Overall, 21% of offspring had occasional parental tobacco exposure at ages 15–17, 11% at ages 20–24, and 6% at age 29; proportions of offspring who had daily parental tobacco exposure at ages 15–17, 20–24 and 29 were 20%, 32% and 17% respectively. For cannabis, 30% of offspring had occasional parental cannabis exposure at age 15–17, 54% at age 20–24, and 16% at age 29; proportions of offspring who had daily parental cannabis exposure at ages 15–17, 20–24 and 29 were 3%, 6% and 5% respectively.

**Table 1:** Characteristics of 1030 children born to 665 parents in the Victorian Intergenerational Health Cohort Study (data imputed and N estimated from proportions).

Study variable	Frequency	
	n	%
**Outcomes**		
Preterm birth	70	6.8
Low birth weight	55	5.4
Small for gestational age	63	6.1
**Preconception exposures**		
Parent tobacco use: 15–17 years		
None	601	58.3
Occasional/weekly	219	21.3
Daily	210	20.4
Parent tobacco use: 20–24 years		
None	587	57.0
Occasional/weekly	118	11.5
Daily	324	31.5
Parent tobacco use: 29 years		
None	791	76.8
Occasional/weekly	64	6.2
Daily	175	16.9
Parent cannabis use: 15–17 years		
None	695	67.5
Occasional/weekly	309	30.0
Daily	26	2.6
Parent cannabis use: 20–24 years		
None	404	39.3
Occasional/weekly	559	54.3
Daily	66	6.4
Parent cannabis use: 29 years		
None	822	79.8
Occasional/weekly	159	15.5
Daily	49	4.7
**Covariates**		
Parent sex: females	609	59.1
Offspring sex: females	519	50.4
Grandparent highest level of education		
Did not complete high school	340	33.0
Completed high school	339	32.9
Completed university	351	34.1
Grandparental divorce	180	17.6
Grandparental daily tobacco use	369	35.8
Parental adolescent mental health problems^a^	396	38.4
Low family SES^b^	211	20.5

^a^Depression/anxiety assessed prospectively through the revised Clinical Interview Schedule.

^b^Home postcode at study entry within the lowest 20% of the Index of Relative Socio-economic Disadvantage.

**Figure 2:**
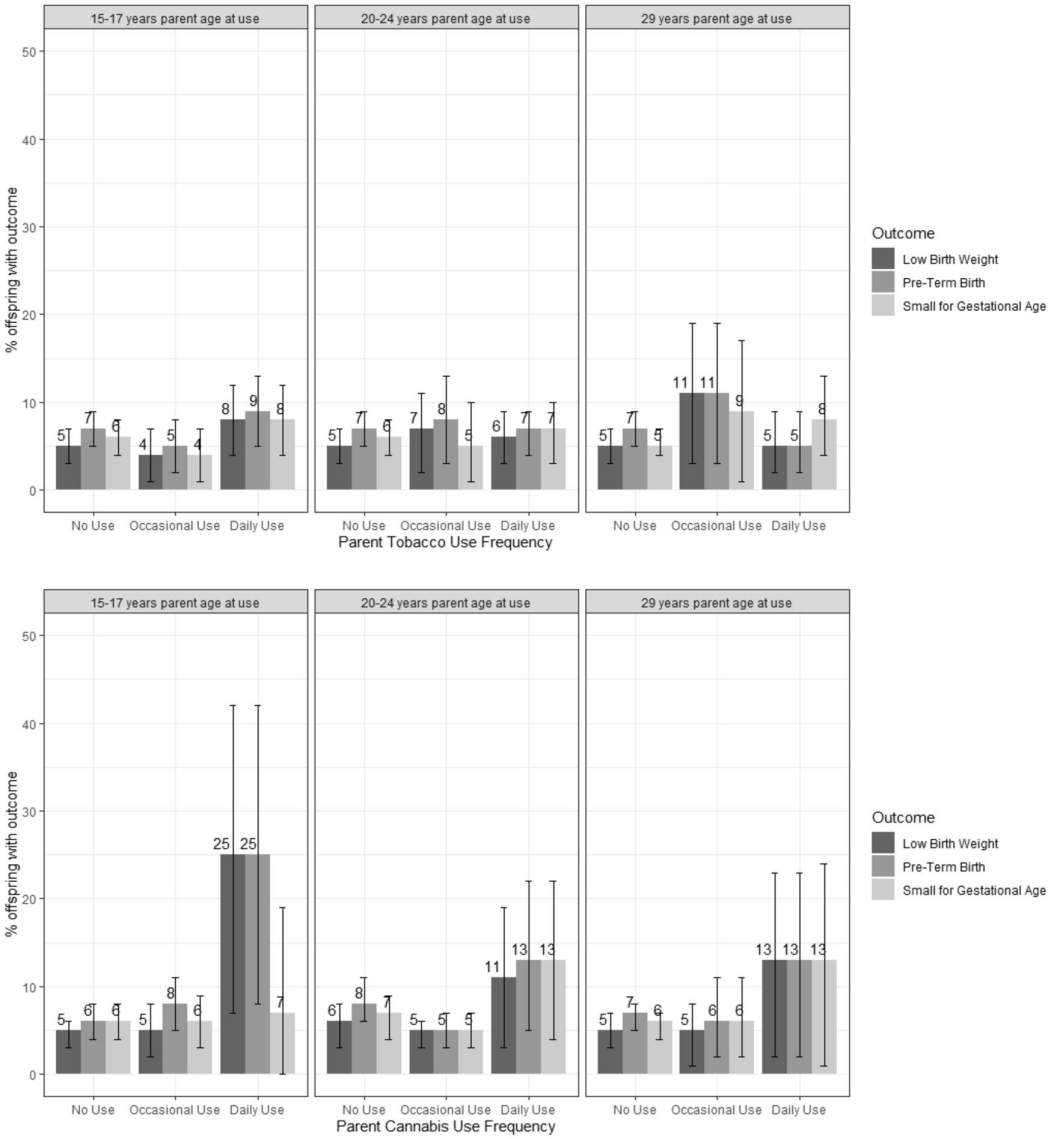
Estimate proportion of children with each birth outcome by frequency of parent substance use in 1030 children born to 665 parents In the VIHCS study (imputed data).

Figure 2 shows the estimated proportions of all birth outcomes in the imputed data, by timing and frequency of parental substance use. Of the offspring whose parents reported daily cannabis use at age 15–17, 25–26% were born either PTB or LBW. In contrast, PTB or LBW were observed in only 11–13% of offspring whose parents reported daily cannabis use at either age 20–24 or age 29.

### Preconception parent substance use and preterm birth (PTB)

There was strong evidence that daily cannabis use at age 15–17 was associated with over a six-fold increase in the odds of offspring PTB, after adjusting for offspring sex and baseline sociodemographic factors, parent sex, family SES, parent mental health at baseline and concurrent tobacco use (aOR 6.65, 95% CI 1.92, 23.09) (Table 2, Fig. 2). Similar associations were observed for the continuous outcome of gestational age in linear models with offspring whose parents reported daily cannabis use at age 15–17 being born on average more than 1 week earlier than those whose parents reported no cannabis use (mean difference − 1.49 weeks, 95% CI − 3.00, 0.03; Supplementary Table 2). There was no evidence that these associations attenuated after further adjustment for any periconceptional/antenatal use (Supplementary Appendix 2, Table 2).

**Table 2:** Logistic regression analysis of relationship between tobacco/cannabis use frequency at age 15–17, 20–24 and 29, and preterm birth in 1030 children born to 665 parents (OR = odds ratio, CI = confidence interval).

Preconception substance use	Offspring preterm birth					
	Unadjusted			Adjusted^a^		
	OR	(95% CI)	*p*	OR	(95% CI)	*p*
**Parent age 15–17 years**						
Tobacco use						
None	1.00			1.00		
Occasional/weekly	0.73	(0.31–1.72)	0.47	0.53	(0.22–1.33)	0.18
Daily	1.32	(0.66–2.64)	0.43	0.71	(0.32–1.60)	0.41
Cannabis use						
None	1.00			1.00		
Occasional/weekly	1.42	(0.74–2.70)	0.29	1.65	(0.80–3.41)	0.18
Daily	5.52	(1.76–17.32)	0.00	6.65	(1.92–23.09)	0.00
**Parent age 20–24 years**						
Tobacco use						
None	1.00			1.00		
Occasional/weekly	1.14	(0.43–3.01)	0.80	1.34	(0.50–3.60)	0.56
Daily	0.97	(0.51–1.82)	0.92	0.79	(0.33–1.88)	0.59
Cannabis use						
None	1.00			1.00		
Occasional/weekly	0.57	(0.31–1.07)	0.08	0.37	(0.18–0.76)	0.01
Daily	1.64	(0.69–3.91)	0.26	0.58	(0.16–2.08)	0.41
**Parent age 29 years**						
Tobacco use						
None	1.00			1.00		
Occasional/weekly	1.65	(0.64–4.25)	0.30	1.84	(0.70–4.79)	0.21
Daily	0.77	(0.32–1.86)	0.57	0.62	(0.23–1.70)	0.35
Cannabis use						
None	1.00			1.00		
Occasional/weekly	0.96	(0.45–2.05)	0.92	0.71	(0.26–1.93)	0.51
Daily	2.02	(0.67–6.06)	0.21	1.47	(0.39–5.53)	0.57

^a^All adjusted models adjusted for baseline/adolescent covariates: parent sex, offspring sex, family SES, grandparent education level, grandparent smoking status, adolescent mental health, and concurrent use of cannabis for models where tobacco is the exposure and vice versa. Adjusted models at age 20–24 years also include adjustment for frequency of use of the substance at age 15–17 years. Adjusted models at age 29 years models also include adjustment for frequency of use of the substance at age 15–17 and age 20–24 years.

### Preconception parent substance use and low birth weight (LBW)

As with preterm birth, there was strong evidence that daily cannabis use at age 15–17 was associated with an almost sixfold increase in odds of offspring being born LBW (aOR 5.84, 95% CI 1.70–20.08) (Table 3). Similarly, in the linear analysis of continuous outcomes, parent’s daily cannabis use in adolescence was associated with an average reduction in birth weight by 400 g after adjustment for all confounders stated above (mean difference − 0.40 kg, 95% CI − 0.85, 0.06; Supplementary Table 3). There was no evidence that these associations attenuated after further adjustment for any periconceptional/antenatal use (Supplementary Appendix 2, Table 2).

**Table 3:** Logistic regression analysis of relationship between tobacco/cannabis use frequency at age 15–17, 20–24 and 29, and low birthweight in 1030 children born to 665 parents (OR = odds ratio, CI = confidence interval).

Preconception substance use	Offspring low birthweight					
	Unadjusted			Adjusted^a^		
	OR	(95% CI)	*p*	OR	(95% CI)	*p*
**Parent age 15–17 years**						
Tobacco use						
None	1.00			1.00		
Occasional/weekly	0.86	(0.34–2.20)	0.75	0.78	(0.29–2.12)	0.63
Daily	1.65	(0.77–3.51)	0.19	1.31	(0.55–3.12)	0.55
Cannabis use						
None	1.00			1.00		
Occasional/weekly	1.06	(0.50–2.26)	0.87	1.01	(0.45–2.28)	0.98
Daily	6.60	(2.09–20.89)	0.00	5.84	(1.70–20.08)	0.01
**Parent age 20–24 years**						
Tobacco use						
None	1.00			1.00		
Occasional/weekly	1.43	(0.48–4.25)	0.52	1.63	(0.53–4.98)	0.39
Daily	1.34	(0.67–2.68)	0.41	1.12	(0.41–3.07)	0.83
Cannabis use						
None	1.00			1.00		
Occasional/weekly	0.79	(0.39–1.61)	0.52	0.56	(0.23–1.35)	0.20
Daily	2.01	(0.71–5.67)	0.19	0.83	(0.20–3.50)	0.80
**Parent age 29 years**						
Tobacco use						
None	1.00			1.00		
Occasional/weekly	2.30	(0.81–6.52)	0.12	2.25	(0.60–8.39)	0.23
Daily	1.12	(0.46–2.75)	0.81	0.73	(0.24–2.21)	0.58
Cannabis use						
None	1.00			1.00		
Occasional/weekly	0.92	(0.38–2.25)	0.86	0.63	(0.18–2.19)	0.47
Daily	2.68	(0.88–8.19)	0.08	1.66	(0.40–6.97)	0.49

^a^All adjusted models adjusted for baseline/adolescent covariates: parent sex, offspring sex, family SES, grandparent education level, grandparent smoking status, adolescent mental health, and concurrent use of cannabis for models where tobacco is the exposure and vice versa. Adjusted models at age 20–24 years also include adjustment for frequency of use of the substance at age 15–17 years. Adjusted models at age 29 years models also include adjustment for frequency of use of the substance at age 15–17 and age 20–24 years.

There was no evidence of an association with preconception tobacco use or with parent occasional/weekly cannabis use at any preconception phase on binary LBW (see Table 3). In analysis of the continuous birth weight outcome there was weak evidence that parental daily tobacco use at age 20–24 was associated with an increase in offspring birth weight, after adjustment for all confounders (mean difference 0.14 kg, 95% CI 0.01, 0.27; Supplementary Table 3).

### Preconception parent substance use and small for gestational age (SGA)

There was little evidence for an association between parental tobacco or cannabis use at any age and offspring being small for gestational age (Table 4), and similar results were observed in the linear regression analysis (Supplementary Table 4).

**Table 4:** Logistic regression analysis of relationship between tobacco/cannabis use frequency at age 15–17, 20–24 and 29, and small for gestational age in 1030 children born to 665 parents (OR = odds ratio, CI = confidence interval).

Preconception substance use	Offspring small for gestational age					
	Unadjusted			Adjusted^a^		
	OR	(95% CI)	*p*	OR	(95% CI)	*p*
**Parent age 15–17 years**						
Tobacco use						
None	1.00			1.00		
Occasional/weekly	0.66	(0.28–1.58)	0.35	0.62	(0.24–1.59)	0.32
Daily	1.31	(0.67–2.54)	0.43	1.39	(0.59–3.27)	0.46
Cannabis use						
None	1.00			1.00		
Occasional/weekly	1.07	(0.57–2.02)	0.84	0.99	(0.45–2.19)	0.98
Daily	1.16	(0.20–6.87)	0.87	0.82	(0.10–6.52)	0.85
**Parent age 20–24 years**						
Tobacco use						
None	1.00			1.00		
Occasional/weekly	0.83	(0.31–2.22)	0.71	0.94	(0.33–2.68)	0.90
Daily	1.09	(0.58–2.06)	0.78	0.97	(0.36–2.60)	0.96
Cannabis use						
None	1.00			1.00		
Occasional/weekly	0.68	(0.36–1.28)	0.23	0.64	(0.30–1.37)	0.25
Daily	2.06	(0.84–5.06)	0.12	1.90	(0.57–6.25)	0.29
**Parent age 29 years**						
Tobacco use						
None	1.00			1.00		
Occasional/weekly	1.80	(0.66–4.94)	0.25	2.50	(0.82–7.62)	0.11
Daily	1.64	(0.84–3.21)	0.15	1.94	(0.81–4.68)	0.14
Cannabis use						
None	1.00			1.00		
Occasional/weekly	1.12	(0.51–2.49)	0.77	0.97	(0.33–2.81)	0.95
Daily	2.33	(0.79–6.89)	0.13	1.91	(0.52–7.03)	0.33

^a^All adjusted models adjusted for baseline/adolescent covariates: parent sex, offspring sex, family SES, grandparent education level, grandparent smoking status, adolescent mental health, and concurrent use of cannabis for models where tobacco is the exposure and vice versa. Adjusted models at age 20–24 years also include adjustment for frequency of use of the substance at age 15–17 years. Adjusted models at age 29 years models also include adjustment for frequency of use of the substance at age 15–17 and age 20–24 years.

### Post-hoc calculation of PAF

The adjusted attributable fraction for the association between daily cannabis use age 15–17 and preterm birth was 20%, and 10% for low birthweight (see Appendix 1 for full details).

## Discussion

We found striking associations between higher frequency parental cannabis use in adolescence and later birth outcomes of offspring; daily use was associated with a more than six-fold increase in the odds of premature birth. These associations remained after adjustment for a broad range of confounders. There was a similar association with offspring low birth weight but no effect on being born small for gestational age, suggesting that the association with low birthweight reflects gestational age at birth, rather than adverse effects on fetal growth. In contrast, there was no consistent association between preconception tobacco use and offspring birth outcomes, and no dose–response was observed at lower levels of cannabis frequency. The adjusted attributable fractions for the association with preterm birth was 20%; if replicated in larger samples, this suggests that eliminating daily cannabis use in adolescents could reduce rates of preterm birth.

Rates of prematurity and low birth weight were consistent with estimated rates for these outcomes in Australia in the 2000s^9,43^. Rates of adolescent cannabis use were similar to those of adolescents in the Australian population in the 1990s^44^ (the period in which the parents in VAHCs were adolescent).

There is a range of possible explanations for the observed associations. Unmeasured confounding from parental exposure to stress and social disadvantage cannot be excluded, despite controlling for grandparental education and baseline mental health as indicators of stress and disadvantage. Adolescent cannabis use may also increase risk of offspring preterm birth through a number of mediating psychosocial pathways that warrant further investigation in larger samples. For example, there is evidence that heavy or regular cannabis use may influence later health risks such as other substance use^45^, poor nutrition^46^, and intimate partner violence^47^, each in turn associated with heightened risk of offspring preterm birth^48–51^.

An alternative potential explanation is continuity of use from adolescence to the periconception and antenatal period, with potential effects on spermatogenesis and the gestational environment^52^. We found that adjustment for any periconceptional or antenatal use did not attenuate findings, but it is plausible that heavier dose or longer duration of periconceptional or antenatal use may mediate preconception associations^53^. However, our findings were not necessarily consistent with this explanation. Effect sizes for offspring birth outcomes were larger for distal daily cannabis use at ages 15–17 than for the more proximal (to the time of conception, which was at age 29 years or older) daily cannabis use at age 20–24.

An intriguing further possibility is the persistence of the effect of preconception adolescent daily cannabis use on reproductive biology. Animal studies have previously shown links between cannabis use and gonadal function and both male and female fertility^54^, and dysregulation of the endocannabinoid system has been linked to pregnancy outcomes^55^. There is some evidence that exposures during prepuberty may be particularly influential on reproductive development in males^37^. In animal studies parental cannabis use predicts altered gamete epigenetic marks involving both DNA methylation and histone modification, with phenotypic change in the next generation^56,57^, and recent evidence has indicated cannabinoid exposure affects human sperm methylation^34^. Such mechanisms could go partway to explaining our results.

This the first prospective study of associations between parent preconception tobacco and cannabis use and birth outcomes. Strengths of the study include the repeated assessment of cannabis and tobacco use across 15 years from adolescence to young adulthood before pregnancy. Alongside these there are certain limitations. Some analyses may be limited by low power; power precluded consideration of differential effects by sex of parent or offspring, which remains an important question for future research. Effect sizes were large, with confidence intervals well above one for binary outcomes and supplementary linear analyses complementing the main findings, but replication of these results in larger intergenerational samples or pooled samples with similarly strong longitudinal designs is now needed. Substance use was assessed by self-report, with potential for reporting bias, though there is evidence that maternal report of tobacco use during pregnancy is valid^58^, and rates of cannabis use in this sample were consistent with rates amongst adolescents in the general population^44^. Prospective substance use data were only available for the parent who was recruited to the original VAHCS cohort.

We accounted for a range of potential confounding variables, but potential for unmeasured confounding remains. VAHCS maintained a high retention rate, and 85% of those with live births during screening participated in VIHCS. Moreover, the retained and participating samples were broadly representative of the baseline VAHCS and eligible VIHCS samples on measured baseline characteristics. Nonetheless, as with all cohort studies, potential for selection bias due to differences on unmeasured characteristics remains. Similarly, we accounted for potential biases due to missing data using multiple imputation with a rich covariate and auxiliary variable set. Finally, it is common in Australia for cannabis to be smoked with tobacco^59^ we cannot rule out an effect of an interaction with the combined use of these drugs.

Frequent adolescent cannabis use was most common in males^60^, a group largely overlooked in relation to public health messaging regarding substance use and birth outcomes where the focus has been predominantly on antenatal tobacco and alcohol use in women^16,17^. Our findings require replication in larger and diverse samples, along with investigation of the potential mechanisms of transmission. Whether mediated by direct and enduring effects on parental reproductive biology, continued use into the periconceptional period, or other psychosocial pathways, our findings support expansion of the developmental origins of disease hypothesis to include the time before conception and provide a compelling new rationale for reducing frequent use during adolescence, particularly in an era where cannabis legalisation in many jurisdictions is increasing cannabis availability.

## Supplementary Information

Supplementary Information.
