# Supplementary material for: Cannabis and tobacco use prior to pregnancy and subsequent offspring birth outcomes: a 20-year intergenerational prospective cohort study
Source: Sci Rep. 2021 Aug 19;11:16826. doi: 10.1038/s41598-021-95460-2 (PMC8376878; doi:10.1038/s41598-021-95460-2)
Supplement: Supplementary file 1 — Supplementary Information. [file 41598_2021_95460_MOESM1_ESM.docx]

**Appendices and Supplementary Materials for**

**Cannabis and tobacco use prior to pregnancy and subsequent offspring birth outcomes: a 20-year intergenerational prospective cohort study**

Lindsey A Hines PhD*, Centre for Academic Mental Health and MRC Integrative Epidemiology Unit, University of Bristol; Bristol, UK

Elizabeth A Spry PhD, Centre for Social and Early Emotional Development, Faculty of Health, Deakin University, and Centre for Adolescent Health, Murdoch Children’s Research Institute; Melbourne, AUSTRALIA

Margarita Moreno-Betancur PhD, University of Melbourne and Murdoch Children’s Research Institute; Melbourne, AUSTRALIA

Hanafi Mohamad Husin MBiostat, Centre for Adolescent Health, Murdoch Children’s Research Institute; Melbourne, AUSTRALIA

Denise Becker MSc, Biostatistics Unit, Faculty of Health, Deakin University, Melbourne, AUSTRALIA

Melissa Middleton MSc, Clinical Epidemiology & Biostatistics Unit, Murdoch Children’s Research Institute; Melbourne, AUSTRALIA

Jeffrey M Craig PhD, Centre for Molecular and Medical Research, Deakin University School of Medicine, Geelong, AUSTRALIA

Lex W Doyle MD, **Department of Obstetrics and Gynaecology, The Royal Women’s Hospital, University of Melbourne; Clinical Sciences,** Murdoch Children’s Research Institute; and **Department of Paedatrics, University of Melbourne;** Melbourne, AUSTRALIA

Craig A Olsson PhD, Centre for Adolescent Health, Murdoch Children’s Research Institute, and Centre for Social and Early Emotional Development, Faculty of Health, Deakin University, Melbourne, AUSTRALIA

George Patton, Centre for Adolescent Health, Murdoch Children’s Research Institute; Melbourne MRCPsych, AUSTRALIA

**Table of contents**

| Page | Item |
| --- | --- |
| 3 | Supplementary figure 1: Sampling and ascertainment of VIHCS, by sex of VAHCS cohort parent |
| 4 | Appendix 1: Methodology for calculating Population-Attributable Fraction |
| 5 | Supplementary Table 1. Proportion of missing observed data for the 1030 children born to 665 parents in the Victorian Intergenerational Health Cohort Study |
| 6-7 | Supplementary table 2: Linear regression analysis of relationship between tobacco/cannabis use frequency at age 15-17, 20-24 and 29, and week of birth |
| 8-9 | Supplementary table 3: Linear regression analysis of relationship between tobacco/cannabis use frequency at age 15-17, 20-24 and 29, and birth weight |
| 10-11 | Supplementary table 4: Linear regression analysis of relationship between tobacco/cannabis use frequency at age 15-17, 20-24 and 29, and size at gestational age |
| 12-14 | Appendix 2: Analyses adjusted for tobacco/cannabis use during pregnancy |


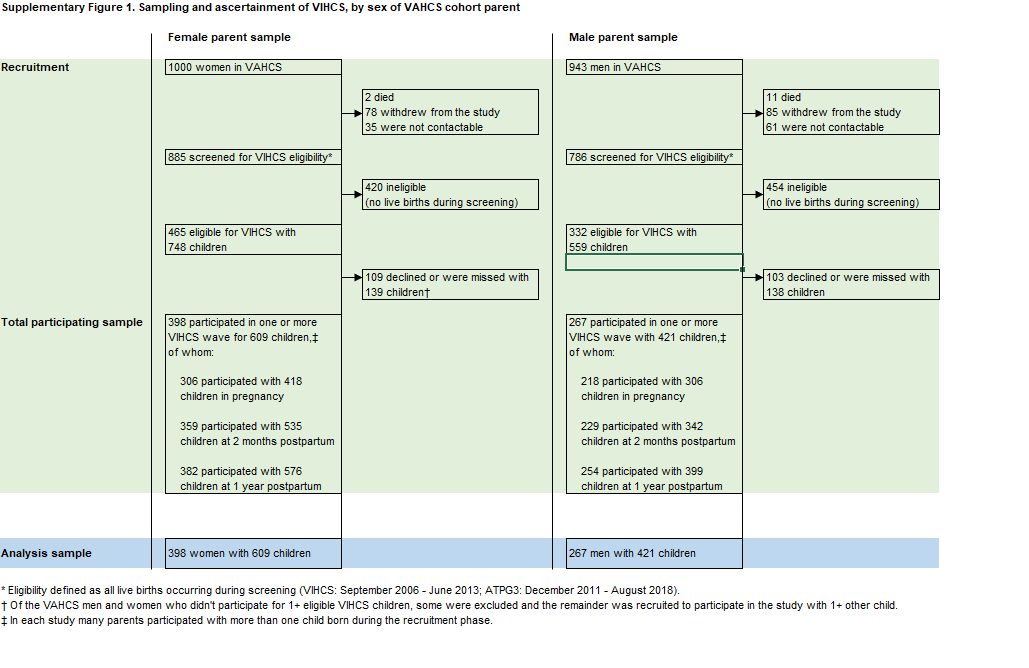


Appendix 1: Methodology for calculating Population-Attributable Fraction

Population-attributable fractions (PAFs) and their 95% confidence intervals were estimated from fully adjusted models via the punaf command in Stata, using the methods recommended by Greenland and Drescher (1993) for cohort and cross-sectional studies. Assuming that estimates from these models provide a good approximation of the true causal effects, the PAF represents the percentage change in premature birth rates that could be achieved if frequent cannabis use were entirely eliminated from the population. It is a comparison of this ideal scenario (in which no parent used cannabis frequently) with the real-world scenario (in which cannabis was used frequently by some parents but not others). In both ideal and real-world scenarios, the distribution of the covariates is as observed in our data.

Estimates were obtained by pooling results across imputed datasets using Rubin’s rules.

Newson R. Attributable and unattributable risks and fractions and other scenario comparisons. Stata J 2013;13:672–98

Greenland S, Drescher K. Maximum likelihood estimation of the attributable fraction from logistic models. Biometrics 1993;49: 865–72.

Rubin DB. Multiple Imputation for Nonresponse in Surveys. Hoboken, NJ, USA: John Wiley & Sons, Inc.; 1987.

Supplementary Table 1. Proportion of missing observed data for the 1030 children born to 665 parents in the Victorian Intergenerational Health Cohort Study

| **Study Variable** | | **Missing** |
| --- | --- | --- |
|  |  | % |
| ***Outcomes*** | |  |
| Preterm birth | | 7.1 |
| Low birth weight | | 7.8 |
| Small for gestational age | | 8.5 |
| ***Preconception Exposures*** | |  |
| Tobacco | |  |
| Wave 2 | | 10.5 |
| Wave 3 | | 9.2 |
| Wave 4 | | 9.2 |
| Wave 5 | | 9.7 |
| Wave 6 | | 14.2 |
| Wave 7 | | 9.4 |
| Wave 8 | | 6.1 |
| Wave 9 | | 3.2 |
| Cannabis | |  |
| Wave 2 | | 11.9 |
| Wave 3 | | 10.6 |
| Wave 4 | | 10.0 |
| Wave 5 | | 11.7 |
| Wave 6 | | 14.9 |
| Wave 7 | | 7.1 |
| Wave 8 | | 6.3 |
| Wave 9 | | 3.5 |
| ***Potential mediators/confounders*** | |  |
| Parent sex | | 0.0 |
| Offspring sex | | 0.3 |
| Maternal antenatal tobacco/cannabis use | | 34.6 |
| Paternal antenatal tobacco/cannabis use | | 38.1 |
| Parental adolescent mental health problems | | 0.0 |
| Grandparental daily tobacco use | | 1.4 |
| Grandparent highest level of education | | 1.0 |
| Grandparental divorce | | 0.0 |
| Family SES | | 1.3 |

Supplementary table 2: Linear regression analysis of relationship between tobacco/cannabis use frequency at age 15-17, 20-24 and 29, and week of birth in 1030 children born to 665 parents

| **Preconception substance use** | **Offspring Gestational Age (week)** | | | | | |
| --- | --- | --- | --- | --- | --- | --- |
|  | Unadjusted | | | Adjusted^a^ | | |
|  | beta | (95% CI) | p | beta | (95% CI) | p |
|  | | | | | | |
| **Parent age 15-17 years** | | | | | | |
| *Tobacco use* | | | | | | |
| None | 0.00 |  |  | 0.00 |  |  |
| Occasional/weekly | 0.17 | (-0.15 to 0.50) | 0.29 | 0.28 | (-0.07 to 0.62) | 0.11 |
| Daily | -0.01 | (-0.39 to 0.38) | 0.98 | 0.22 | (-0.19 to 0.64) | 0.29 |
| *Cannabis use* | | | | | | |
| None | 0.00 |  |  | 0.00 |  |  |
| Occasional/weekly | -0.02 | (-0.31 to 0.27) | 0.90 | -0.11 | (-0.44 to 0.22) | 0.50 |
| Daily | -1.38 | (-2.94 to 0.17) | 0.08 | -1.49 | (-3.00 to 0.03) | 0.05 |
|  | | | | | | |
| **Parent age 20-24 years** | | | | | | |
| *Tobacco use* | | | | | | |
| None | 0.00 |  |  | 0.00 |  |  |
| Occasional/weekly | 0.20 | (-0.20 to 0.61) | 0.33 | 0.18 | (-0.25 to 0.60) | 0.41 |
| Daily | 0.14 | (-0.17 to 0.44) | 0.37 | 0.22 | (-0.16 to 0.59) | 0.25 |
| *Cannabis use* | | | | | | |
| None | 0.00 |  |  | 0.00 |  |  |
| Occasional/weekly | 0.22 | (-0.07 to 0.50) | 0.13 | 0.22 | (-0.09 to 0.52) | 0.16 |
| Daily | -0.39 | (-0.99 to 0.22) | 0.21 | -0.08 | (-0.86 to 0.69) | 0.83 |
|  | | | | | | |
| **Parent age 29 years** | | | | | | |
| *Tobacco use* | | | | | | |
| None | 0.00 |  |  | 0.00 |  |  |
| Occasional/weekly | -0.25 | (-0.75 to 0.25) | 0.32 | -0.32 | (-0.84 to 0.20) | 0.23 |
| Daily | 0.31 | (-0.07 to 0.69) | 0.10 | 0.35 | (-0.09 to 0.79) | 0.12 |
| *Cannabis use* | | | | | | |
| None | 0.00 |  |  | 0.00 |  |  |
| Occasional/weekly | 0.13 | (-0.25 to 0.50) | 0.50 | 0.26 | (-0.16 to 0.68) | 0.22 |
| Daily | -0.51 | (-1.19 to 0.18) | 0.14 | -0.27 | (-1.04 to 0.51) | 0.50 |

^a^ All adjusted models adjusted for baseline/adolescent covariates: parent sex, offspring sex, family SES, grandparent education level, grandparent smoking status, adolescent mental health, and concurrent use of cannabis for models where tobacco is the exposure and vice versa. Adjusted models at age 20-24 years also include adjustment for frequency of use of the substance at age 15-17 years. Adjusted models at age 29 years models also include adjustment for frequency of use of the substance at age 15-17 and age 20-24 years.

Supplementary table 3: Linear regression analysis of relationship between tobacco/cannabis use frequency at age 15-17, 20-24 and 29, and birth weight in 1030 children born to 665 parents

| **Preconception substance use** | **Offspring Birth Weight (kg)** | | | | | |
| --- | --- | --- | --- | --- | --- | --- |
|  | Unadjusted | | | Adjusted^a^ | | |
|  | beta | (95% CI) | p | beta | (95% CI) | p |
|  | | | | | | |
| **Parent age 15-17 years** | | | | | | |
| *Tobacco use* | | | | | | |
| None | 0.00 |  |  | 0.00 |  |  |
| Occasional/weekly | 0.10 | (-0.01 to 0.21) | 0.08 | 0.12 | (0.00 to 0.23) | 0.05 |
| Daily | -0.04 | (-0.16 to 0.09) | 0.57 | 0.00 | (-0.14 to 0.13) | 0.95 |
| *Cannabis use* | | | | | | |
| None | 0.00 |  |  | 0.00 |  |  |
| Occasional/weekly | 0.00 | (-0.09 to 0.10) | 0.95 | -0.01 | (-0.12 to 0.09) | 0.79 |
| Daily | -0.41 | (-0.86 to 0.05) | 0.08 | -0.40 | (-0.85 to 0.06) | 0.09 |
|  | | | | | | |
| **Parent age 20-24 years** | | | | | | |
| *Tobacco use* | | | | | | |
| None | 0.00 |  |  | 0.00 |  |  |
| Occasional/weekly | 0.07 | (-0.08 to 0.21) | 0.35 | 0.06 | (-0.09 to 0.21) | 0.41 |
| Daily | 0.08 | (-0.02 to 0.18) | 0.13 | 0.14 | (0.01 to 0.27) | 0.03 |
| *Cannabis use* | | | | | | |
| None | 0.00 |  |  | 0.00 |  |  |
| Occasional/weekly | 0.09 | (0.00 to 0.18) | 0.05 | 0.09 | (-0.02 to 0.20) | 0.10 |
| Daily | -0.12 | (-0.32 to 0.08) | 0.24 | -0.06 | (-0.29 to 0.17) | 0.60 |
|  | | | | | | |
| **Parent age 29 years** | | | | | | |
| *Tobacco use* | | | | | | |
| None | 0.00 |  |  | 0.00 |  |  |
| Occasional/weekly | -0.08 | (-0.28 to 0.11) | 0.38 | -0.15 | (-0.35 to 0.05) | 0.14 |
| Daily | 0.03 | (-0.09 to 0.15) | 0.59 | 0.01 | (-0.13 to 0.15) | 0.89 |
| *Cannabis use* | | | | | | |
| None | 0.00 |  |  | 0.00 |  |  |
| Occasional/weekly | -0.03 | (-0.15 to 0.09) | 0.61 | 0.00 | (-0.13 to 0.13) | 0.96 |
| Daily | -0.14 | (-0.38 to 0.09) | 0.23 | -0.08 | (-0.33 to 0.17) | 0.52 |

^a^ All adjusted models adjusted for baseline/adolescent covariates: parent sex, offspring sex, family SES, grandparent education level, grandparent smoking status, adolescent mental health, and concurrent use of cannabis for models where tobacco is the exposure and vice versa. Adjusted models at age 20-24 years also include adjustment for frequency of use of the substance at age 15-17 years. Adjusted models at age 29 years models also include adjustment for frequency of use of the substance at age 15-17 and age 20-24 years.

Supplementary table 4: Linear regression analysis of relationship between tobacco/cannabis use frequency at age 15-17, 20-24 and 29, and size at gestational age in 1030 children born to 665 parents

| **Preconception substance use** | **Offspring Gestational Age Birthweight Z-Score** | | | | | |
| --- | --- | --- | --- | --- | --- | --- |
|  | Unadjusted | | | Adjusted^a^ | | |
|  | beta | (95% CI) | p | beta | (95% CI) | p |
|  | | | | | | |
| **Parent age 15-17 years** | | | | | | |
| *Tobacco use* | | | | | | |
| None | 0.00 |  |  | 0.00 |  |  |
| Occasional/weekly | 0.14 | (-0.07 to 0.35) | 0.19 | 0.13 | (-0.09 to 0.35) | 0.26 |
| Daily | -0.09 | (-0.30 to 0.12) | 0.42 | -0.12 | (-0.37 to 0.12) | 0.33 |
| *Cannabis use* | | | | | | |
| None | 0.00 |  |  | 0.00 |  |  |
| Occasional/weekly | 0.02 | (-0.15 to 0.20) | 0.79 | 0.03 | (-0.18 to 0.24) | 0.78 |
| Daily | -0.30 | (-0.94 to 0.33) | 0.34 | -0.22 | (-0.88 to 0.45) | 0.52 |
|  | | | | | | |
| **Parent age 20-24 years** | | | | | | |
| *Tobacco use* | | | | | | |
| None | 0.00 |  |  | 0.00 |  |  |
| Occasional/weekly | 0.05 | (-0.22 to 0.33) | 0.69 | 0.06 | (-0.24 to 0.35) | 0.70 |
| Daily | 0.09 | (-0.09 to 0.27) | 0.31 | 0.20 | (-0.04 to 0.44) | 0.11 |
| *Cannabis use* | | | | | | |
| None | 0.00 |  |  | 0.00 |  |  |
| Occasional/weekly | 0.11 | (-0.06 to 0.28) | 0.21 | 0.10 | (-0.11 to 0.31) | 0.36 |
| Daily | -0.14 | (-0.50 to 0.23) | 0.46 | -0.08 | (-0.49 to 0.32) | 0.68 |
|  | | | | | | |
| **Parent age 29 years** | | | | | | |
| *Tobacco use* | | | | | | |
| None | 0.00 |  |  | 0.00 |  |  |
| Occasional/weekly | -0.10 | (-0.45 to 0.25) | 0.58 | -0.19 | (-0.56 to 0.18) | 0.31 |
| Daily | -0.07 | (-0.28 to 0.13) | 0.49 | -0.13 | (-0.40 to 0.13) | 0.32 |
| *Cannabis use* | | | | | | |
| None | 0.00 |  |  | 0.00 |  |  |
| Occasional/weekly | -0.11 | (-0.32 to 0.10) | 0.31 | -0.11 | (-0.35 to 0.12) | 0.34 |
| Daily | -0.13 | (-0.49 to 0.24) | 0.49 | -0.07 | (-0.46 to 0.32) | 0.73 |

^a^ All adjusted models adjusted for baseline/adolescent covariates: parent sex, offspring sex, family SES, grandparent education level, grandparent smoking status, adolescent mental health, and concurrent use of cannabis for models where tobacco is the exposure and vice versa. Adjusted models at age 20-24 years also include adjustment for frequency of use of the substance at age 15-17 years. Adjusted models at age 29 years models also include adjustment for frequency of use of the substance at age 15-17 and age 20-24 years.

Supplementary table 5: Complete case data analysis - Logistic regression analysis of relationship between tobacco/cannabis use frequency at age 15-17, 20-24 and 29, and pre-term birth, low birth weight and size at gestational age in 1030 children born to 665 parents

|  | **Outcomes** | | | | | | | | |
| --- | --- | --- | --- | --- | --- | --- | --- | --- | --- |
|  | Pre-term birth | | | Low birth weight | | | SGA | | |
|  | OR | (95% CI) | p | OR | (95% CI) | p | OR | (95% CI) | p |
| **Early Adolescence** | | | | | | | | | |
| *Tobacco use* | | | | | | | | | |
| No/Occasional use | 1.00 |  |  | 1.00 |  |  | 1.00 |  |  |
| Weekly | 0.43 | (0.13 to 1.46) | 0.18 | 1.22 | (0.43 to 3.46) | 0.70 | 0.75 | (0.27 to 2.14) | 0.59 |
| Daily | 0.68 | (0.21 to 2.25) | 0.53 | 1.85 | (0.59 to 5.77) | 0.29 | 2.36 | (0.70 to 7.96) | 0.17 |
| *Cannabis use* | | | | | | | | | |
| No/ Occasional use | 1.00 |  |  | 1.00 |  |  | 1.00 |  |  |
| Weekly | 1.04 | (0.34 to 3.15) | 0.95 | 0.53 | (0.16 to 1.74) | 0.30 | 0.48 | (0.15 to 1.58) | 0.23 |
| Daily | 9.79 | (2.25 to 42.65) | 0.00 | 7.38 | (1.97 to 27.57) | 0.00 | 0.42 | (0.03 to 5.00) | 0.49 |
| **Late Adolescence** | | | | | | | | | |
| *Tobacco use* | | | | | | | | | |
| No/Occasional use | 1.00 |  |  | 1.00 |  |  | 1.00 |  |  |
| Weekly | 1.80 | (0.63 to 5.15) | 0.28 | 2.46 | (0.75 to 8.02) | 0.14 | 1.21 | (0.39 to 3.76) | 0.74 |
| Daily | 0.61 | (0.22 to 1.70) | 0.35 | 0.84 | (0.26 to 2.72) | 0.77 | 0.89 | (0.25 to 3.08) | 0.85 |
| *Cannabis use* | | | | | | | | | |
| No/Occasional use | 1.00 |  |  | 1.00 |  |  | 1.00 |  |  |
| Weekly | 0.40 | (0.17 to 0.94) | 0.04 | 0.49 | (0.16 to 1.56) | 0.23 | 0.55 | (0.23 to 1.30) | 0.17 |
| Daily | 0.37 | (0.06 to 2.48) | 0.31 | 1.13 | (0.20 to 6.32) | 0.89 | 3.46 | (0.67 to 17.85) | 0.14 |
| **Adult** | | | | | | | | | |
| *Tobacco use* | | | | | | | | | |
| No/Occasional use | 1.00 |  |  | 1.00 |  |  | 1.00 |  |  |
| Weekly | 2.32 | (0.65 to 8.32) | 0.19 | 3.93 | (0.76 to 20.39) | 0.10 | 3.67 | (1.01 to 13.33) | 0.05 |
| Daily | 0.54 | (0.17 to 1.71) | 0.30 | 0.76 | (0.24 to 2.43) | 0.64 | 1.89 | (0.73 to 4.87) | 0.19 |
| *Cannabis use* | | | | | | | | | |
| No/Occasional use | 1.00 |  |  | 1.00 |  |  | 1.00 |  |  |
| Weekly | 0.19 | (0.02 to 1.59) | 0.13 | 0.21 | (0.03 to 1.69) | 0.14 | 0.78 | (0.14 to 4.54) | 0.79 |
| Daily | 1.41 | (0.18 to 11.23) | 0.75 | 0.98 | (0.11 to 9.01) | 0.99 | 2.27 | (0.31 to 16.82) | 0.42 |

Supplementary Table 6: Logistic regression analysis of relationship between tobacco/cannabis use frequency at age 15-17, 20-24 and 29, and preterm birth in 1030 children born to 665 parents (OR = odds ratio, CI=confidence interval) **imputed generating a summary exposure variable (This approach involved setting data to missing for all exposure waves, for any participant missing 1+ wave of exposure data)**

| **Preconception substance use** | **Offspring Preterm Birth** | | | | | |
| --- | --- | --- | --- | --- | --- | --- |
|  | Unadjusted | | | Adjusted^a^ | | |
|  | OR | (95% CI) | p | OR | (95% CI) | p |
|  | | | | | | |
| **Parent age 15-17 years** | | | | | | |
| *Tobacco use* | | | | | | |
| None | 1.00 |  |  | 1.00 |  |  |
| Occasional/weekly | 0.56 | (0.21 to 1.47) | 0.24 | 0.42 | (0.16 to 1.16) | 0.09 |
| Daily | 1.28 | (0.65 to 2.52) | 0.47 | 0.71 | (0.29 to 1.75) | 0.46 |
| *Cannabis use* | | | | | | |
| None | 1.00 |  |  | 1.00 |  |  |
| Occasional/weekly | 1.02 | (0.49 to 2.14) | 0.96 | 1.27 | (0.54 to 2.98) | 0.59 |
| Daily | 4.55 | (1.67 to 12.39) | 0.00 | 5.61 | (1.66 to 18.98) | 0.01 |
|  | | | | | | |
| **Parent age 20-24 years** | | | | | | |
| *Tobacco use* | | | | | | |
| None | 1.00 |  |  | 1.00 |  |  |
| Occasional/weekly | 1.11 | (0.41 to 2.95) | 0.84 | 1.22 | (0.45 to 3.34) | 0.70 |
| Daily | 0.90 | (0.48 to 1.71) | 0.75 | 0.64 | (0.24 to 1.70) | 0.37 |
| *Cannabis use* | | | | | | |
| None | 1.00 |  |  | 1.00 |  |  |
| Occasional/weekly | 0.57 | (0.30 to 1.09) | 0.09 | 0.37 | (0.17 to 0.80) | 0.01 |
| Daily | 1.68 | (0.71 to 3.99) | 0.24 | 0.55 | (0.14 to 2.17) | 0.39 |
|  | | | | | | |
| **Parent age 29 years** | | | | | | |
| *Tobacco use* | | | | | | |
| None | 1.00 |  |  | 1.00 |  |  |
| Occasional/weekly | 1.44 | (0.55 to 3.80) | 1.44 | 1.74 | (0.63 to 4.78) | 0.28 |
| Daily | 0.74 | (0.30 to 1.80) | 0.74 | 0.60 | (0.21 to 1.71) | 0.34 |
| *Cannabis use* | | | | | | |
| None | 1.00 |  |  | 1.00 |  |  |
| Occasional/weekly | 0.97 | (0.45 to 2.05) | 0.93 | 0.62 | (0.22 to 1.76) | 0.37 |
| Daily | 1.90 | (0.59 to 6.09) | 0.28 | 1.03 | (0.21 to 5.03) | 0.97 |

^a^ All adjusted models adjusted for baseline/adolescent covariates: parent sex, grandparent education level, grandparent smoking status, adolescent mental health, and concurrent use of cannabis for models where tobacco is the exposure and vice versa. Adjusted models at age 20-24 years also include adjustment for frequency of use of the substance at age 15-17 years. Adjusted models at age 29 years models also include adjustment for frequency of use of the substance at age 15-17 and age 20-24 years.

Supplementary Table 7: Logistic regression analysis of relationship between tobacco/cannabis use frequency at age 15-17, 20-24 and 29, and low birthweight in 1030 children born to 665 parents (OR = odds ratio, CI=confidence interval) **imputed generating a summary exposure variable (This approach involved setting data to missing for all exposure waves, for any participant missing 1+ wave of exposure data)**

| **Preconception substance use** | **Offspring Low Birthweight** | | | | | |
| --- | --- | --- | --- | --- | --- | --- |
|  | Unadjusted | | | Adjusted^a^ | | |
|  | OR | (95% CI) | p | OR | (95% CI) | p |
|  | | | | | | |
| **Parent age 15-17 years** | | | | | | |
| *Tobacco use* | | | | | | |
| None | 1.00 |  |  | 1.00 |  |  |
| Occasional/weekly | 1.04 | (0.40 to 2.71) | 0.94 | 1.01 | (0.38 to 2.67) | 0.99 |
| Daily | 1.73 | (0.81 to 3.71) | 0.16 | 1.47 | (0.53 to 4.09) | 0.45 |
| *Cannabis use* | | | | | | |
| None | 1.00 |  |  | 1.00 |  |  |
| Occasional/weekly | 0.68 | (0.27 to 1.71) | 0.41 | 0.60 | (0.21 to 1.66) | 0.32 |
| Daily | 5.53 | (1.89 to 16.15) | 0.00 | 4.51 | (1.21 to 16.77) | 0.02 |
|  | | | | | | |
| **Parent age 20-24 years** | | | | | | |
| *Tobacco use* | | | | | | |
| None | 1.00 |  |  | 1.00 |  |  |
| Occasional/weekly | 1.42 | (0.47 to 4.27) | 0.53 | 1.35 | (0.46 to 3.98) | 0.58 |
| Daily | 1.31 | (0.64 to 2.68) | 0.46 | 0.86 | (0.32 to 2.27) | 0.75 |
| *Cannabis use* | | | | | | |
| None | 1.00 |  |  | 1.00 |  |  |
| Occasional/weekly | 0.81 | (0.38 to 1.72) | 0.58 | 0.62 | (0.24 to 1.63) | 0.33 |
| Daily | 2.28 | (0.85 to 6.12) | 0.10 | 0.94 | (0.18 to 4.76) | 0.94 |
|  | | | | | | |
| **Parent age 29 years** | | | | | | |
| *Tobacco use* | | | | | | |
| None | 1.00 |  |  | 1.00 |  |  |
| Occasional/weekly | 2.10 | (0.71 to 6.24) | 0.18 | 1.92 | (0.51 to 7.32) | 0.34 |
| Daily | 1.02 | (0.41 to 2.54) | 0.97 | 0.69 | (0.22 to 2.17) | 0.52 |
| *Cannabis use* | | | | | | |
| None | 1.00 |  |  | 1.00 |  |  |
| Occasional/weekly | 0.99 | (0.42 to 2.36) | 0.98 | 0.58 | (0.16 to 2.04) | 0.39 |
| Daily | 2.52 | (0.77 to 8.33) | 0.13 | 1.07 | (0.16 to 7.30) | 0.95 |

^a^ All adjusted models adjusted for baseline/adolescent covariates: parent sex, grandparent education level, grandparent smoking status, adolescent mental health, and concurrent use of cannabis for models where tobacco is the exposure and vice versa. Adjusted models at age 20-24 years also include adjustment for frequency of use of the substance at age 15-17 years. Adjusted models at age 29 years models also include adjustment for frequency of use of the substance at age 15-17 and age 20-24 years.

Supplementary Table 8: Logistic regression analysis of relationship between tobacco/cannabis use frequency at age 15-17, 20-24 and 29, and small for gestational age in 1030 children born to 665 parents (OR = odds ratio, CI=confidence interval) **imputed generating a summary exposure variable (This approach involved setting data to missing for all exposure waves, for any participant missing 1+ wave of exposure data)**

| **Preconception substance use** | **Offspring Small for Gestational Age** | | | | | |
| --- | --- | --- | --- | --- | --- | --- |
|  | Unadjusted | | | Adjusted^a^ | | |
|  | OR | (95% CI) | p | OR | (95% CI) | p |
|  | | | | | | |
| **Parent age 15-17 years** | | | | | | |
| *Tobacco use* | | | | | | |
| None | 1.00 |  |  | 1.00 |  |  |
| Occasional/weekly | 0.69 | (0.27 to 1.74) | 0.43 | 0.75 | (0.29 to 1.94) | 0.55 |
| Daily | 1.30 | (0.68 to 2.49) | 0.43 | 1.93 | (0.77 to 4.80) | 0.16 |
| *Cannabis use* | | | | | | |
| None | 1.00 |  |  | 1.00 |  |  |
| Occasional/weekly | 0.68 | (0.33 to 1.41) | 0.29 | 0.51 | (0.20 to 1.31) | 0.16 |
| Daily | 0.81 | (0.10 to 6.59) | 0.84 | 0.48 | (0.05 to 4.74) | 0.53 |
|  | | | | | | |
| **Parent age 20-24 years** | | | | | | |
| *Tobacco use* | | | | | | |
| None | 1.00 |  |  | 1.00 |  |  |
| Occasional/weekly | 0.93 | (0.36 to 2.43) | 0.88 | 0.96 | (0.35 to 2.62) | 0.94 |
| Daily | 1.04 | (0.56 to 1.90) | 0.91 | 0.83 | (0.31 to 2.22) | 0.71 |
| *Cannabis use* | | | | | | |
| None | 1.00 |  |  | 1.00 |  |  |
| Occasional/weekly | 0.66 | (0.35 to 1.27) | 0.21 | 0.75 | (0.35 to 1.58) | 0.45 |
| Daily | 2.06 | (0.84 to 5.01) | 0.11 | 2.98 | (0.65 to 13.60) | 0.16 |
|  | | | | | | |
| **Parent age 29 years** | | | | | | |
| *Tobacco use* | | | | | | |
| None | 1.00 |  |  | 1.00 |  |  |
| Occasional/weekly | 1.75 | (0.67 to 4.55) | 0.25 | 2.52 | (0.86 to 7.42) | 0.09 |
| Daily | 1.53 | (0.79 to 2.98) | 0.21 | 1.89 | (0.79 to 4.52) | 0.15 |
| *Cannabis use* | | | | | | |
| None | 1.00 |  |  | 1.00 |  |  |
| Occasional/weekly | 1.01 | (0.46 to 2.23) | 0.98 | 0.88 | (0.31 to 2.50) | 0.81 |
| Daily | 1.92 | (0.66 to 5.58) | 0.23 | 1.47 | (0.36 to 6.02) | 0.59 |

^a^ All adjusted models adjusted for baseline/adolescent covariates: parent sex, grandparent education level, grandparent smoking status, adolescent mental health, and concurrent use of cannabis for models where tobacco is the exposure and vice versa. Adjusted models at age 20-24 years also include adjustment for frequency of use of the substance at age 15-17 years. Adjusted models at age 29 years models also include adjustment for frequency of use of the substance at age 15-17 and age 20-24 years.

**Appendix 2: Tobacco/Cannabis exposure during pregnancy**

Periconceptional/antenatal maternal and paternal smoking were measured through the items “Just before finding out you/your partner were/was pregnant, did you smoke cigarettes?” (mothers and fathers) and “Shortly after finding out you were pregnant (say, a couple of weeks), how many cigarettes did you smoke?” (mothers only). Periconceptional/antenatal cannabis use was measured through the items “Just before finding out you/your partner were/was pregnant, how often did you use [cannabis]?” (mothers and fathers) and “Shortly after finding out you were pregnant (say, a couple of weeks), how often did you use [cannabis]?” (mothers only). These four variables were combined into measures of periconceptional/antenatal maternal or paternal tobacco or cannabis use and dichotomised into either none or some.

Appendix 2 Table 1: Frequency of antenatal tobacco/cannabis use in 1030 children born to 665 parents

| **Study Variable** | | **Frequency** | |
| --- | --- | --- | --- |
|  |  | n | % |
| Maternal antenatal tobacco/cannabis use | | 209 | 20.3 |
| Paternal antenatal tobacco/cannabis use | | 274 | 26.6 |

Appendix 2 Table 2: Logistic regression analysis adjusted for antenatal tobacco/cannabis use of relationship between tobacco/cannabis use frequency at age 15-17, 20-24 and 29, and birth outcomes in 1030 children born to 665 parents (OR = odds ratio, CI=confidence interval)

| **Preconception substance use** |  | | | | | |  | | | | |
| --- | --- | --- | --- | --- | --- | --- | --- | --- | --- | --- | --- |
|  | **Offspring Preterm Birth** | | | **Offspring Low Birthweight** | | | **Offspring Small for Gestational Age** | | | | |
|  | AOR | (95% CI) | p | AOR | (95% CI) | p | AOR | | (95% CI) | p | |
| **Parent age 15-17 years** | | | | | | |  | | | | |
| *Tobacco use* | | | | | | |  | | | | |
| None | 1.00 |  |  | 1.00 |  |  | 1.00 | | | | |
| Occasional/weekly | 0.56 | (0.22 to 1.39) | 0.21 | 0.78 | (0.28 to 2.18) | 0.64 | 0.55 | (0.20 to 1.47) | | | 0.23 |
| Daily | 0.78 | (0.33 to 1.83) | 0.56 | 1.29 | (0.51 to 3.27) | 0.59 | 1.10 | (0.46 to 2.66) | | | 0.83 |
| *Cannabis use* | | | | | | |  | | | | |
| None | 1.00 |  |  | 1.00 |  |  | 1.00 | | | | |
| Occasional/weekly | 1.70 | (0.81 to 3.55) | 0.16 | 1.03 | (0.45 to 2.36) | 0.95 | 0.93 | (0.42 to 2.06) | | | 0.86 |
| Daily | 6.90 | (1.89 to 25.20) | 0.00 | 5.59 | (1.57 to 19.92) | 0.01 | 0.73 | (0.09 to 6.03) | | | 0.77 |
|  | | | | | | |  | | | | |
| **Parent age 20-24 years** | | | | | | |  | | | | |
| *Tobacco use* | | | | | | |  | | | | |
| None | 1.00 |  |  | 1.00 |  |  | 1.00 | | | | |
| Occasional/weekly | 1.32 | (0.47 to 3.68) | 0.59 | 1.54 | (0.48 to 4.97) | 0.47 | 0.85 | (0.28 to 2.54) | | | 0.77 |
| Daily | 0.83 | (0.33 to 2.13) | 0.70 | 1.12 | (0.38 to 3.28) | 0.84 | 0.74 | (0.26 to 2.15) | | | 0.58 |
| *Cannabis use* | | | | | | |  | | | | |
| None | 1.00 |  |  | 1.00 |  |  | 1.00 | | | | |
| Occasional/weekly | 0.37 | (0.18 to 0.77) | 0.01 | 0.55 | (0.22 to 1.37) | 0.20 | 0.60 | (0.28 to 1.31) | | | 0.20 |
| Daily | 0.60 | (0.16 to 2.22) | 0.45 | 0.83 | (0.19 to 3.58) | 0.80 | 1.68 | (0.48 to 5.86) | | | 0.41 |
|  | | | | | | |  | | | | |
| **Parent age 29 years** | | | | | | |  | | | | |
| *Tobacco use* | | | | | | |  | | | | |
| None | 1.00 |  |  | 1.00 |  |  | 1.00 | | | | |
| Occasional/weekly | 1.87 | (0.63 to 5.53) | 0.26 | 2.13 | (0.46 to 9.88) | 0.33 | 2.16 | (0.60 to 7.77) | | | 0.24 |
| Daily | 0.65 | (0.22 to 1.95) | 0.44 | 0.72 | (0.21 to 2.41) | 0.59 | 1.60 | (0.61 to 4.22) | | | 0.34 |
| *Cannabis use* | | | | | | |  | | | | |
| None | 1.00 |  |  | 1.00 |  |  | 1.00 | | | | |
| Occasional/weekly | 0.74 | (0.28 to 1.99) | 0.55 | 0.67 | (0.19 to 2.29) | 0.52 | 0.92 | (0.32 to 2.66) | | | 0.88 |
| Daily | 1.56 | (0.41 to 5.91) | 0.51 | 1.73 | (0.41 to 7.32) | 0.45 | 1.76 | (0.47 to 6.59) | | | 0.40 |

Adjusted for antenatal tobacco/cannabis use, parent sex, offspring sex, family SES, grandparent education level, grandparent smoking status, adolescent mental health, and concurrent use of cannabis for models where tobacco is the exposure and vice versa. Adjusted models at age 20-24 years also include adjustment for frequency of use of the substance at age 15-17 years. Adjusted models at age 29 years models also include adjustment for frequency of use of the substance at age 15-17 and age 20-24 years.
